# Increased use of analgesics in midlife women but no association with mental stress: observations from the Prospective Population Study of Women in Gothenburg

**DOI:** 10.1186/s12905-022-01605-4

**Published:** 2022-02-11

**Authors:** Dominique Hange, Gunilla Fernlöf, Cecilia Björkelund, Tove Hedenrud

**Affiliations:** 1 Department of Public Health and Community Medicine/Primary Health Care, School of Public Health and Community Medicine, Institute of Medicine, Sahlgrenska Academy, University of Gothenburg, PO Box 454, 405 30 Gothenburg, Sweden; 2 Research, Education, Development & Innovation, Primary Health Care, Region Västra Götaland, Sweden; 3 Medicine Use & Pharmaceutical Policy, School of Public Health and Community Medicine, Institute of Medicine, University of Gothenburg, Gothenburg, Sweden

**Keywords:** Analgesics, Mental stress, Non-steroidal anti-inflammatory medicine, Population study, Women

## Abstract

**Background:**

The study is part of the ongoing Prospective Population Study of Women in Gothenburg, Sweden, initiated in 1968–1969 with the aim of characterising a total population of women who were representative of middle-aged females. The aim of the present study was to investigate the prevalence of actual analgesic use (prescribed and self-medication) and the possible association with perceived mental stress among women aged 38 and 50 years, respectively, in the Population Study of Women.

**Methods:**

Two different cohorts of population-based samples of 38- and 50-year-old women examined in 2004–2005 and 2016–2017, respectively, were eligible participants. The women were representative for their age cohort at the time of the examinations. Use of medicines and especially analgesics, as well as perceived mental stress, was registered. Changes in medicine use among 38- and 50-year-old women between 2004 and 2005 and 38- and 50-year-old women in 2016–2017 were studied. Data were analysed using logistic regression. Use of analgesics and mental stress were analysed controlling for lifestyle factors, use of other medicines and pain.

**Results:**

The overall sample size across the time periods was 1,073 individuals. The frequency of analgesic use in 38- and 50-year-old women was about 26% in 2004–2005 and 58% in 2016–2017. 28% of women who reported high mental stress in 2004–2005 used analgesics, compared to 60% in 2016–2017. There were no associations between self-perceived mental stress and the use of analgesics.

**Conclusion:**

The higher use of analgesics among midlife women in 2016–2017 is in line with global findings and could be due to increased availability in Sweden of over the counter medicines. The impact of mental stress on analgesic use found previously by other researchers was not confirmed. However, medicine use as a potential coping strategy is an important public health issue that needs to be further explored.

## Background

Sales of analgesics are increasing worldwide, where the United States, China and Brazil are the top three countries (2020) with regard to revenue in the analgesic segments [1]. Analgesics have a prominent position within the context of self-medication, in line with the global trend where more and more medicines are being sold without prescription [2]. In Europe (2017), the sales of analgesic products bought over-the-counter (OTC) showed Italy as the market leader followed by Germany, with Sweden in seventh place [1]. An earlier international comparison showed that the highest per-capita consumption was observed in Sweden with 147 Standard Units per capita per year, an increasing trend from 1986 [3]. In Sweden, the availability of OTC analgesics has increased during the last decade due to a new reform allowing them to be sold in non-pharmacy outlets [4]. This reform also led to an increased number of pharmacies, which further increased the availability of OTC medicines. In a population-based study, about 10% (N = 2594) reported a higher use of OTC medicines due to the increased availability [5]. In most countries, the prevalence of self-medicated analgesic use is higher in women than men [6]. Thus, a Danish population study found that 27% (N = 22,199) of the women and 18% (N = 23,080) of the men aged 18–45 years had a regular monthly use of analgesic tablets [7]. Similar gender differences have been seen in Sweden [8].

The main reason for using analgesics is pain and women generally have a higher prevalence of many pain disorders as well as chronic pain [9]. Self-perceived poor health can also explain much of analgesic use. Other important factors for analgesic use are sleeping problems and health care utilisation [10]. The use of analgesic has been shown to be higher among smokers, and obese adults with medium or high socio- economic status and lower in adults performing more than 2 h/week of physical exercise [11]. Patients with chronic pain can use alcohol together with opioids as treatment [12]. Pain severity has been shown to be the strongest independent predictor of sleep problems [13], which in turn can lead to higher use of analgesic.

Findings from the 90 s showed that the use of tranquilizers, hypnotics, and analgesics could be associated with stressors such as unemployment and conflicts with a spouse [14]. Koushede et al. could later show an association between mental stress and OTC analgesic use, both in men and women 25–44 years old (N = 4739) [15]. Using analgesics for emotional modulation/sedation have also been shown to have a strong association with greater distress and depression [16]. During the last decades an increase of perception of mental stress from around 25 to 75% has been registered in the female middle-aged population in Sweden [17]. There are suggestions that some medicine use may be a behaviour reflecting a general coping strategy to overcome daily stressors outside therapeutic indications [18]. This type of behaviour regarding medicine use is important to study, since it may lead to interactions with prescribed medicines and have an impact on health and well-being. The aim of the present study was to study secular trends of analgesic use and investigate the prevalence of actual analgesic use (prescribed and self-medication) and the possible association with perceived mental stress among women aged 38 and 50 years, respectively, in the Population Study of Women in 2004–2005 and 2016–2017.

## Methods

This observational study uses data from two different cohorts of population-based samples of 38- and 50-year-old women, recruited in 2004–2005 and 2016–2017 from the Prospective Population Study of Women in Gothenburg, Sweden (PSWG) [19]. The present study used cross-sectional data from the two consecutive examinations for investigating the prevalence and secular trends of actual analgesic use (prescribed and self- medication) and the possible association with self-perceived mental stress in middle-aged women.

## Characteristics of participants

In 1968–1969, 1462 women aged 38, 46, 50, 54 and 60 participated in the PSWG [19]. The selection of women was based on their date of birth to make the sample representative. After that, follow up examinations were conducted in 1974–1975, 1980–1981, 1992–1993, 2000–2001, 2004–2005, 2005–2006 and in 2016–2017. The participation rate has been high throughout all years, i.e., around 90% at the three first examinations and 70% in 1992–1993, 71% in 2000–2001 and 72% in 2005–2006. Details of the sampling procedure and participation rates at earlier examinations have been presented elsewhere [19, 20]. Physical examinations were conducted according to the same protocol at all examinations.

**Fig. 1:**
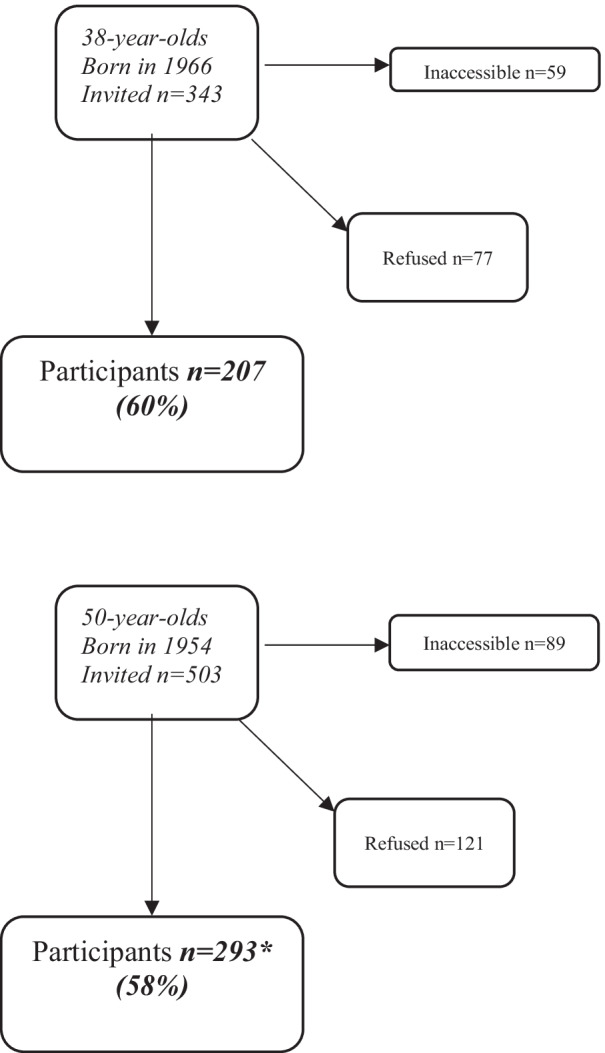
Flow chart of the 2004–2005 examination in the Population Study of Women in Gothenburg. Seven women were excluded because of language difficulties. * 51 of these participated also in 1992–1993

In 2004–2005 a new sample of 38- and 50-year-old women was invited, in which women born in 1966 were examined for the first time. Some of the women born in 1954 had participated in the 1992–1993 examination and the remaining were newly recruited to reach representativeness. A total of 343 women aged 38 and 503 aged 50 were invited to a free health examination. The sample was obtained from the Revenue Office Register, based on the womens’ date of birth. The survey was performed over a six-month period. The women were contacted by post and followed up with a phone call, when the phone number was available. A total of 500 women (207 38-year-olds and 293 50-year-olds) accepted the invitation (73% and 70%, respectively of those reachable by telephone) [21] (Fig. 1).

**Fig. 2:**
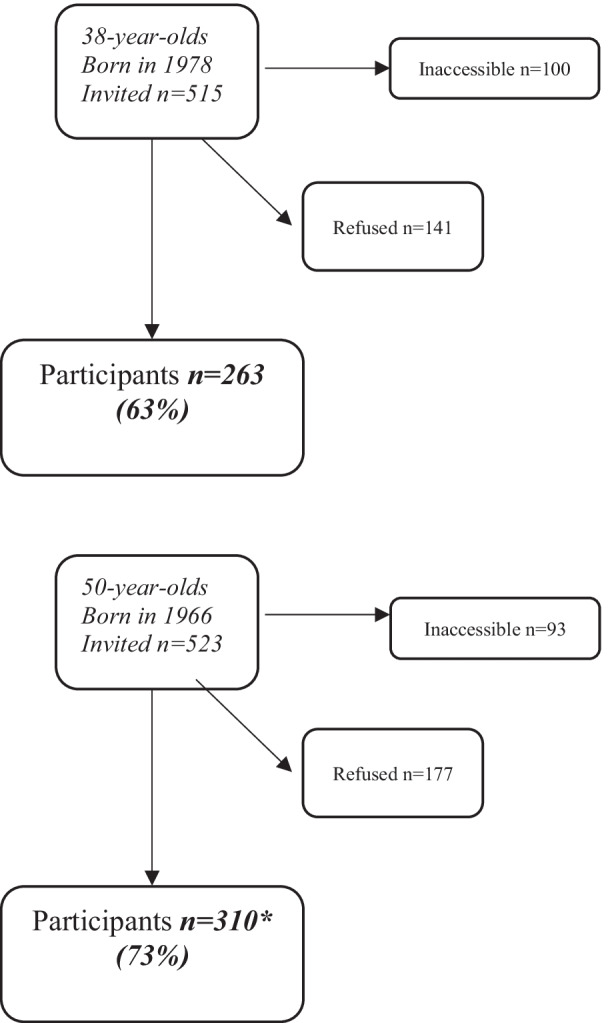
Flow chart of the 2016–2017 examination in the Population Study of Women in Gothenburg. *155 of these participated also in 2004–2005

In 2016–2017 women born in 1978 were examined for the first time, and a new group of women born in1966 was recruited to reach representativeness, the remainder having participated in 2004–2005. A total of 515 women aged 38 and 523 aged 50 were invited to a free health examination. The sample was obtained from the Revenue Office Register. The survey was performed over an 8-month period. Invitations were sent out by post and followed up with a phone call, when the phone number was available. A total of 573 women (263 38-year-olds and 310 50-year-olds) accepted the invitation (63% and 73% of those reachable per telephone, respectively) [22] (Fig. 2).

For the purpose of the present study, it was decided to increase power to detect differences between 2004–2005 and 2016–2017 cohorts by merging 38-year-olds and 50-year-olds into one group, respectively, in order to obtain a larger group of women at each time point, reaching the number of 500 women in 2004–2005 and 573 in 2016–2017.

Study population characteristics, i.e., social group, smoking, alcohol consumption, leisure time physical activity, and amount of sleep are shown in Table 1.

**Table 1:** Background characteristics of 38-and 50-year-old women in the Population Study of Women in Gothenburg

	2004–2005 n (%)	2016–2017 n (%)
Social group		
Low	134 (26.8)	102 (17.8)
Medium	244 (48.8)	258 (45.0)
High	105 (21.0)	192 (33.5)
Missing	17 (3.4)	21 (3.7)
Education		
Low	232 (46.4)	190 (33.2)
High	261 (52.2)	374 (65.3)
Missing	7 (1.4)	9 (1.6)
Smoking		
Currently	90 (18.0)	60 (10.5)
Never/ever	405 (81.0)	513 (89.5)
Missing	5 (1.0)	–
Alcohol		
Never	39 (7.8)	86 (15.0)
Sometimes	342 (68.4)	393 (68.6)
Regularly	111 (22.2)	92 (16.1)
Missing	8 (1.6)	2 (0.3)
Leisure time exercise		
Less than 4 h/week	94 (18.8)	49 (8.6)
At least 4 h /week	399 (79.8)	522 (91.1)
Missing	7 (1.4)	2 (0.3)
Amount of sleep		
Not enough sleep	213 (42.6)	259 (45.2)
Enough sleep	279 (55.8)	306 (53.4)
Missing	8 (1.6)	8 (1.4)
Total (n)	500	573

### Definition and identification of analgesic use

This study focuses on anti-inflammatory and anti-rheumatic medicines (M01), analgesics (N02), opioids (N02A), other analgesics (N02B), salicylates (N02BA), paracetamol (N02BE) and anti-migraine medicines (N02C). The dependent variable was use or no use of medicines within ATC (Anatomical Therapeutic Chemical) code [23], M01 and/or N02. The nurse noted on a standardised form the name of the product, the ATC code, strength, number of units and whether use was daily or when needed (prn).

Other medicines that were included in the analysis were anti-acid medicines (A02), sedatives (N05C), hypnotics (N05B) and antidepressants (N06A). Both daily use and prn use were included. These were chosen as covariates due to their frequent use in relation to mental stress.

### Definitions of mental stress

The prevalence of mental stress was based on a question describing the extent to which the women had experienced mental stress previously or presently. Participants were asked: “Have you experienced any period of mental stress (1 month or more), and with mental stress we mean that you have been irritable, tense, nervous, anxious, afraid, anguished or unable to sleep, connected with concern for your work, your health, your family or conflict with the people around you (at home, at work)?”.

To describe the prevalence of mental stress, a model with three levels was used:

- Mental stress level 0–1: no mental stress during the last 5 years, including the current period
- Mental stress level 2: occasionally experienced mental stress during the last 5 years
- Mental stress level 3–5: experienced constant or several periods of mental stress during the last 5 years.

The mental stress question was asked in exactly the same way at all follow ups. The question has been used in various epidemiological studies [24, 25] and has been found to be a valid measure of mental stress, compared to other psychometric instruments [26].

### Social group and education

The women reported their occupations and total income status, and this information was used to classify their social group into three categories: high, medium, or low. The categorisation was based primarily on the husband's profession, secondly on the women's own profession, and was carried out in accordance with the Swedish socioeconomic index, that includes the number of years in an occupation in combination with educational level [27]. Low socioeconomic status designated skilled and unskilled workers, intermediate socioeconomic status small-scale employers, officials of lower rank and foreman, high socioeconomic status large-scale employers and officials of high or intermediate rank.

Based on the number of years reported by each woman, higher education was defined as education beyond elementary school (more than 6–7 years).

## Smoking

Smoking habits was defined as whether the woman was a current smoker or not.

### Alcohol consumption

Information regarding alcohol habits was obtained using a standardised structured interview conducted by a physician. All participants were asked to report the frequency of their consumption of three different types of alcoholic drinks, i.e., beer, wine, and spirits. Women were asked: “Do you use beer, wine, and/or spirits” and then to report the frequency according to the following alternatives: 0 = Never; 1 = Earlier, but not during the last 10 years; 2 = Earlier, but not during the last year; 3 = Monthly; 4 = Weekly; 5 = Several times a week; 6 = Daily [28].

For the purpose of this study, we derived three separate categories of consumption of any alcohol, based on the various levels (frequency) were: never (including levels 0, 1 and 2); sometimes (including levels 3 and 4); regularly (including levels 5 and 6).

### Physical activity

Leisure time physical activity (LTPA) was assessed in terms of 4 categories: ‘almost inactive' (reading, watching TV, etc.), ‘some physical activity at least 4 h/week' (cycling, walking, etc.), ‘regular exercise' (tennis, running, heavy gardening, etc.), and ‘regular training and competitive sports' [25]. Physical inactivity was defined as low LTPA, i.e., less than 4 h of LTPA per week versus more.

### Sleep quality

The study question regarding sleep “Is your sleep long enough?” with the response alternatives: Yes or No.

### Pain experience

A general question about pain experience was included in the study: “How much bodily pain have you had during the last 4 weeks?”. The response alternatives were: None, very mild, mild, moderate, severe, very severe. For the purpose of this study the alternatives were classified into three categories; “none”, “very mild and mild”, and “moderate, severe and very severe”.

### Data collection

At the study clinic, a nurse inquired about current medicine use. Participants were asked the following: “Considering any medicine prescribed by a doctor or purchased without a prescription in a pharmacy—what are you currently using?”.

### Statistical methods

Background characteristics and frequency of analgesic use were analysed as proportions for each study year (2004–2005 and 2016–2017). Proportion of analgesic users for each study year was analysed in relation to level of perceived mental stress.

Logistic regression analysis was performed for each study year where analgesic use was the dependent variable. Perceived mental stress, together with age and social group, was entered in the first model. In the second model, the covariates of pain and other medicines were added. Finally, in the third model, lifestyle covariates were added. Associations were reported as odds ratios (OR) with 95% confidence intervals (CI). All analyses were performed with SPSS Statistics, version 25 (IBM Corporation, New York, USA).

## Results

A higher proportion of women belonged to the highest social group in 2016–2017 than in 2004–2005 (Table 1). Leisure time physical activity of at least 4 h/week increased from almost 80% in 2004–2005 to > 90% in 2016–2017. Current smokers decreased from 18 to 10% during the same period. No other significant differences between the two cohorts were seen.

Table 2 shows the different types of analgesics the women used in 2004–2005 and 2016–2017. Overall, there was a higher proportion of women using analgesics in 2016–2017 compared to 2004–2005. For paracetamol, the difference in use was almost threefold between the study years.

**Table 2:** Use of different types of analgesics among 38-and 50-year-old women in 2004–2005 and 2016–2017 in the Population Study of Women, Gothenburg

Type of analgesic	Study year 2004–2005 n (%)	Study year 2016–2017 n (%)
Anti-inflammatory and anti-rheumatic medicines (M01)	63 (12.6)	191 (33.3)
Analgesics (N02)	94 (18.8)	234 (40.8)
Opioids (N02A)	15 (3.0)	13 (2.3)
Other analgesics (N02B)	75 (15.0)	219 (38.2)
Salicylates (N02BA)	23 (4.6)	25 (4.4)
Paracetamol (N02BE)	60 (12.0)	203 (35.4)
Anti-migraine medicines (N02C)	14 (2.8)	12 (2.1)
M01 + N02	132 (26.4)	335 (58.5)
Total (n)	500	573

There were 244 women reporting the highest level of stress in 2004–2005 of which 28.3% used analgesics (M01 + N02). Corresponding figures in 2016–2017 were 319 and 60.5% (Table 3).

**Table 3:** Frequency of use of analgesics (M01 + N02) in 38- and 50-year- old women reporting different levels of self-perceived mental stress in 2004–2005 and 2016–2017 respectively

Stress level	Use of analgesics	
	2004–2005 n (%)	2016–2017 n (%)
No stress (0–1)	35 (27.8)	78 (55.3)
Stress to some extent (2)	25 (21.4)	62 (56.4)
Stress to a high extent (3–5)	69 (28.3)	193 (60.5)
Total	129 (25.8)	333 (58.1)

In the regression analysis with analgesic use as the dependent variable, still no significant associations with perceived mental stress could be shown. In the fully adjusted model for 2004–2005 there was an association between analgesic use and pain during the last 4 weeks, as well as use of anti-acid medicines (Table 4). In 2016–2017, there was an association between use of analgesics and age (Table 5).

**Table 4:** Logistic regression analysis of the relationship between self-perceived stress and use of analgesics in 2004–2005, adjusted for covariates in three models

	Model 1 OR (95% CI)	Model 2 OR (95% CI)	Model 3 OR (95% CI)
Self-perceived mental stress			
No stress	Ref	Ref	Ref
Stress to some extent	0.69 (0.38–1.25)	0.68 (0.37–1.27)	0.65 (0.34–1.22)
Stress to a high extent	1.10 (0.68–1.79)	0.93 (0.55–1.58)	0.91 (0.54–1.56)
Age			
38	Ref	Ref	Ref
50	0.99 (0.96–1.03)	0.98 (0.94–1.01)	0.98 (0.94–1.02)
Social group*			
1	1.62 (0.85–3.11)	1.97 (0.98–3.96)	1.85 (0.90–3.78)
2	1.20 (0.70–2.04)	1.44 (0.81–2.56)	1.41 (0.79–2.52)
3	Ref	Ref	Ref
Education**	1.27 (0.80–2.02)	1.26 (0.77–2.06)	1.34 (0.81–2.22)
Pain during past 4 weeks			
None		Ref	Ref
Very mild or mild		2.16 (1.20–3.90)	2.26 (1.24–4.12)
Moderate/severe/very severe		3.52 (1.92–6.46)	3.73 (2.01–6.93)
Use of “anti-acid medicines”		5.41 (2.27–12.91)	5.56 (2.31–13.39)
Use of sedatives		0.71 (0.12–4.15)	0.69 (0.12–4.07)
Use of hypnotics		0.84 (0.23–3.00)	0.84 (0.24–3.03)
Use of antidepressants		0.94 (0.14–6.24)	0.86 (0.13–5.80)
Smoking			
Never/ever			Ref
Currently			0.77 (0.43–1.40)
Alcohol			
Never			Ref
Sometimes			1.37 (0.57–3.33)
Regularly			1.40 (0.53–3.72)
Leisure time exercise			
At least 4 h/week			Ref
Less than 4 h /week			1.00 (0.58–1.75)
Amount of sleep			
Enough sleep			Ref
Not enough sleep			1.12 (0.71–1.76)
Goodness of fit (chi-square, degrees of freedom; *p* value)	3.746; 8; 0.879	3.615; 8; 0.890	4.438; 8; 0.816

*1. High, large-scale employers and officials of high or intermediate rank. 2. Medium, small-scale employers, officials of lower rank and foreman. 3. Low, skilled and unskilled workers

**Higher education was defined as education beyond elementary school (more than 6–7 years)

**Table 5:** Logistic regression analysis of the relationship between self-perceived stress and use of analgesics in 2016–2017 adjusted for covariates in three models

	Model 1 OR (95% CI)	Model 2 OR (95% CI)	Model 3 OR (95% CI)
Self-perceived mental stress			
No stress	Ref	Ref	Ref
Stress to some extent	0.97 (0.58–1.62)	0.94 (0.56–1.58)	0.91 (0.54–1.55)
Stress to a high extent	1.22 (0.80–1.84)	1.03 (0.67–1.59)	0.98 (0.63–1.53)
Age			
38	Ref	Ref	Ref
50	0.97 (0.94–0.997)	0.96 (0.93–0.99)	0.96 (0.93–0.99)
Social group			
1	1.12 (0.63–1.97)	1.09 (0.61–1.96)	0.98 (0.53–1.82)
2	1.14 (0.68–1.91)	1.07 (0.63–1.81)	1.00 (0.57–1.74)
3	Ref	Ref	Ref
Education	0.99 (0.65–1.51)	0.98 (0.64–1.51)	1.00 (0.64–1.55)
Pain during last 4 weeks			
None		Ref	Ref
Very mild or mild		1.23 (0.80–1.89)	1.16 (0.75–1.79)
Moderate/severe/very severe		1.68 (1.03–2.73)	1.54 (0.93–2.53)
Use of “anti-acid medicines”		1.82 (0.78–4.26)	1.84 (0.78–4.33)
Use of sedatives		1.67 (0.42–6.64)	1.65 (0.41–6.58)
Use of hypnotics		1.44 (0.52–4.02)	1.34 (0.48–3.77)
Use of antidepressants		1.55 (0.83–2.91)	1.59 (0.85–2.99)
Smoking			
Never/ever			Ref
Currently			1.00 (0.54–1.85)
Alcohol			
Never			Ref
Sometimes			1.55 (0.89–2.69)
Regularly			1.36 (0.69–2.68)
Leisure time exercise			
At least 4 h/week			Ref
Less than 4 h /week			0.82 (0.43–1.59)
Amount of sleep			
Enough sleep			Ref
Not enough sleep			1.31 (0.90–1.90)
Goodness of fit (chi-square, degrees of freedom; p-value)	1.818; 7; 0.969	9.822; 8; 0.278	4.315; 8; 0.828

## Discussion

This study used two cohorts of the same ages (38 and 50 years) at a 12-year interval to investigate whether self-perceived mental stress was associated with analgesic use. Our study did not show any associations among women between their use of analgesics and self-perceived mental stress, either in 2004–2005 or in 2016–2017, despite a more than doubled frequency of analgesic use in the later examination, i.e., from 26 to 58%. Thus, we could not confirm findings concerning women, found in Koushede et al.’s study of an association between stress and OTC analgesic use [15]. Their further investigations showed that sense of coherence (SOC) modified the association between perceived stress and medicine use for headache in women [18]. Unfortunately, our study did not include questions about SOC.

The most relevant factor for using analgesic were having pain during the last 4 weeks, both in 2004–2005 and 2016–2017 (only model 2), which could be expected. This also confirms previous Swedish research [10]. Our results could, however, not confirm associations found by Antonov et al. concerning alcohol, smoking or sleeping problems [10]. Wranker et al. showed that earlier cohorts of women used higher amounts of pain killers than the later cohorts and that men with pain used more alcohol than women with pain [29]. Our study could not confirm these findings. Cohort comparisons concerning analgesic use in women have been published earlier. A Finnish survey showed that the use was connected with occupational class, self-rated health and feelings of loneliness [30].

Previous research showed that a generally higher medicine use in women compared to men was attributed to their higher level of physical distress [31] and that college students’ (N = 231) use of OTC medications was associated with self-reported distress [32]. Another study has shown associations between perceived high job stress, measured by Karasek’s job-strain model, and the use of benzodiazepines during the last month, both in women and men [33]. The use of benzodiazepines in our study was too low to allow worthwhile calculations to be performed.

Our study could not confirm previous research indicating that some medicine use may be a behaviour reflecting a general coping strategy to overcome daily stressors, and several explanations for this are possible. Our study used a different questionnaire for mental stress, albeit it has been evaluated [26], and the one used by Koushede also included perceived stress. We had also fewer participants than our Danish colleagues (N = 4739) [15], which could influence the association.

Other authors have pointed out depression, oral pain and physical inactivity as measures having positive associations with self-medication [34, 35]. We did not find any associations between analgesic use and use of antidepressants or level of leisure time physical activity.

The reported usage of anti-inflammatory and anti-rheumatic medicines (M01) was higher in the 2016–2017 examination. National statistics from the Swedish Prescribed Drug Register (SPDR) show that prescriptions of M01 have decreased from 167 patients/1000 inhabitants 35–54 years old (both genders) in 2006 to 124 patients/1000 inhabitants in 2019, and from 189 patients/1000 inhabitants to 143 patients/1000 inhabitants among women during the same years [36]. This suggests that more of the women in the later examination used over-the-counter medicines, which seems reasonable considering the increased availability.

The use of paracetamol increased also in our study, from 12% in 2004–2005 to 35% in 2016–2017. This has been shown by other Swedish researchers, where from 2006 to 2015, the yearly prevalence of paracetamol increased, especially in young females [8]. Another study found that 70% of the population had used paracetamol in the last three months [37].

Every year recommendations and therapy advice to prescribers are presented from Drug and Therapeutics Committee in Västra Götaland Region in a booklet containing prescription guidelines. In 2004–2005, ibuprofen was recommended as the first-line medicine for short-acting pain relief and paracetamol as peripheral analgesics. In 2016–2017, naproxen became the first-line recommendation, but still paracetamol and ibuprofen are recommended [38]. Research has shown that these guidelines are followed to a high extent by primary health care doctors [39].

The re-regulation of the Swedish pharmacy market mentioned earlier has impacted on medicine use in Sweden [40]. How the use of analgesics is affected by contemporary events, such as advertising or that a medicine is withdrawn due to excessive risks, is difficult to ascertain, but there were at least two important events during the time of the study. First, there was a very active advertising with ibuprofen (Ipren) on Swedish television, which began in 1999 and continued until 2011 [41]. Second, the withdrawal of rofecoxib (Vioxx) in 2004 due to risk of cardiovascular events [42] occurred simultaneously with the collection of data for the 2004–2005 population in our study.

Among the advantages of this study are the representative selection of women, based on their date of birth as well as the cross-sectional and longitudinal designs with the same examination protocol across the years. The question concerning mental stress has been used in previous research and found to be a valid measure [26].

There are also some limitations to this study. The number of participants might have been too small to identify potential associations between self-perceived mental stress and analgesic use in women. The women in our study were 38 and 50 years old but using only younger cohorts might have given different results, since other risk perceptions have been shown by young adults when it comes to over-the-counter medicine [43].

Participants were asked about frequency of intake of analgesics and other medicines, both regarding prescription and OTC medicines by the study nurse. The questionnaire regarding medicines was unfortunately not designed in collaboration with pharmacists, which made it difficult to obtain detailed data on the use of medicines. This is something we will consider when planning for the new follow-up of PSWG in a couple of years.

## Conclusion

The secular trends of higher use of analgesics among midlife women in 2016–2017 compared to 2004–2005 is in line with global findings and could be due to increased availability in Sweden of OTC medicines. The impact of mental stress on analgesic use was not confirmed. However, medicine use as a potential coping strategy is an important public health issue that needs to be further explored.

## Data Availability

The data sets used and analysed during the current study are available from the corresponding author on reasonable request.

## Abbreviations

ATC code: Anatomical therapeutic chemical code
LTPA: Leisure time physical activity
OTC: Over-the-counter
prn: When needed
PSWG: Population Study of Women in Gothenburg
SPDR: Swedish prescribed drug register
